# Petrositis caused by fluconazole-resistant candida: case report and literature review

**DOI:** 10.1186/s12879-022-07637-3

**Published:** 2022-07-27

**Authors:** Ling Jin, Shuangxi Liu, Shiwang Tan, Yang Wang, Yumin Zhao, Shaoqing Yu

**Affiliations:** Department of Otolaryngology, Tongji Hospital, School of Medicine, Tongji University, 389 Xincun Road, Putuo, Shanghai, 200065 China

**Keywords:** Petrositis, Gradenigo's syndrome, Candida, Diagnoses, Drug treatment

## Abstract

**Background:**

Petrositis is a rare and fatal complication associated with otitis media. It is most likely caused by bacterial infections, but in some cases it is caused by fungal infections.

**Case study:**

The case in this report is associated with fungal petrositis. The clinical symptoms are: ear pain from chronic otitis media, severe headache, peripheral facial palsy and diplopia. The case was finally confirmed through imaging of middle ear, bacterial culture, pathology, and blood Metagenomic next-generation sequencing (mNGS) test. The patient was treated with sensitive antifungal drugs.

**Conclusion:**

Drug treatment is conservative but efficient method in this case. mNGS can provide pathogenic reference, when antibiotic is not efficient enough for fungal infections or drug-resistant fungal infections cases. This allows we to adjust drug use for the treatment.

## Background

Petrous apex is a part of the temporal bone. Petrositis is a rare complication of otitis media. A search through PubMed with key word petrositis showed that there were only 182 relevant publications in the past 76 years from January 1936 to March 2022. And most of them were only case reports (112 cases). 3 cases were tuberculosis related and only 2 cases were fungal related. (1 case was secondary nasopharyngeal aspergillus and the other case was secondary candidal otitis media). Main clinical symptoms for petrositis are: ear discharge, headache, and abducens nerve palsy. It is also called syndrome of petrous apex. As it was first mentioned by Gradenigo, it is also called Gradenigos syndrome [1]. Data shows that during 2009–2011, there were 5,811,127 Emergency Care visits for acute otitis media or related complications in U.S [2]. But there was only 366 Gradenigos cases reported [2]. The weighted frequency is only 0.006%, [2], that shows Petrositis’ rarity.

Otitis media is a common disease in otolaryngology. With advanced medical tec hnology, complication cases are tremendously reduced. So this can cause some lack of awareness for some physicians., Because the trigeminal ganglion and sixth cranial nerve in the petrosal apex area are only attached to a very thin bone when middle ear infection spreads to the petrous apex, it can cause damage to these nerves easily. Then it causes deep facial pain, diplopia by parlaying the lateral rectus muscle [3]. Due to the widespread use of antibiotics, most symptoms of petrositis are atypical. Under such circumstance, it is difficult to diagnose and treat petrositis. Undiagnosed and inadequately treated petrositis cases can lead to serious complications: meningitis, epidural and intracranial abscesses, cranial nerve palsy, venous sinus thrombosis, subdural abscess, labyrinthitis, even death [4]. The case reported is petrositis caused by drug-resistant fungal infection. So the diagnosis and treatment process were even more difficult and complicated. We haven’t found any similar case after viewing relevant documents.

## Case presentation

**Fig. 1:**
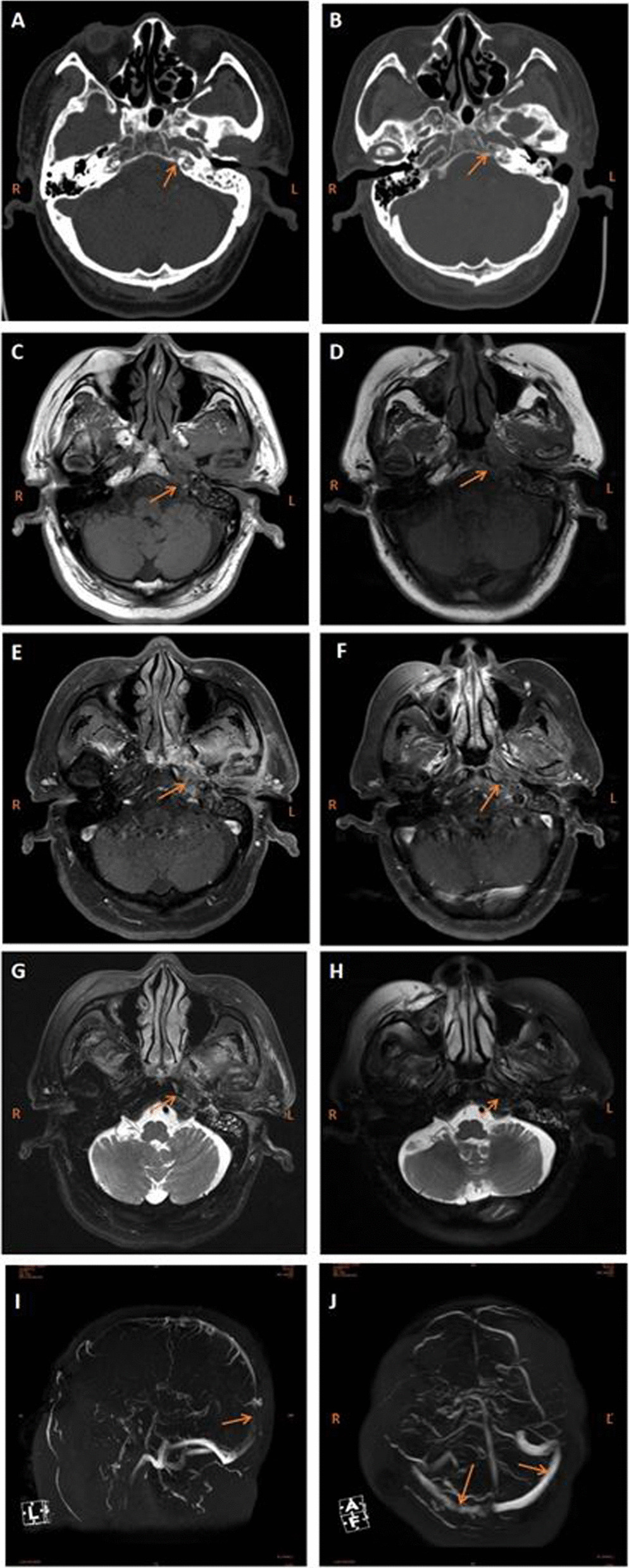
Image of the patient. **A** and **B** Pre-and post-operative CT scan images of the middle ear, showing slightly blurred bone in the apical region. **C** and **D** Pre-and post-operative middle ear transverse position T1WI MRI, showing low signal in the apex of petrous part. **E** and **F** Pre-and post-operative middle ear transverse position T1WI + FS enhancement MRI, showing moderate enhancement in the apex of petrous part, with no significant preoperative or postoperative changes. **G** and **H** Pre-and post-operative middle ear transverse position T1WI + FS MRI, showing slightly higher signal in the apex of petrous part with blurred boundaries. **I** and **J** Cranial MRV, showing irregular wall of posterior superior sagittal sinus, narrow lumen, low blood flow signal, considerd thrombosis; Left sigmoid sinus normal, Decreased blood flow signal in the right sigmoid sinus

**Fig. 2:**
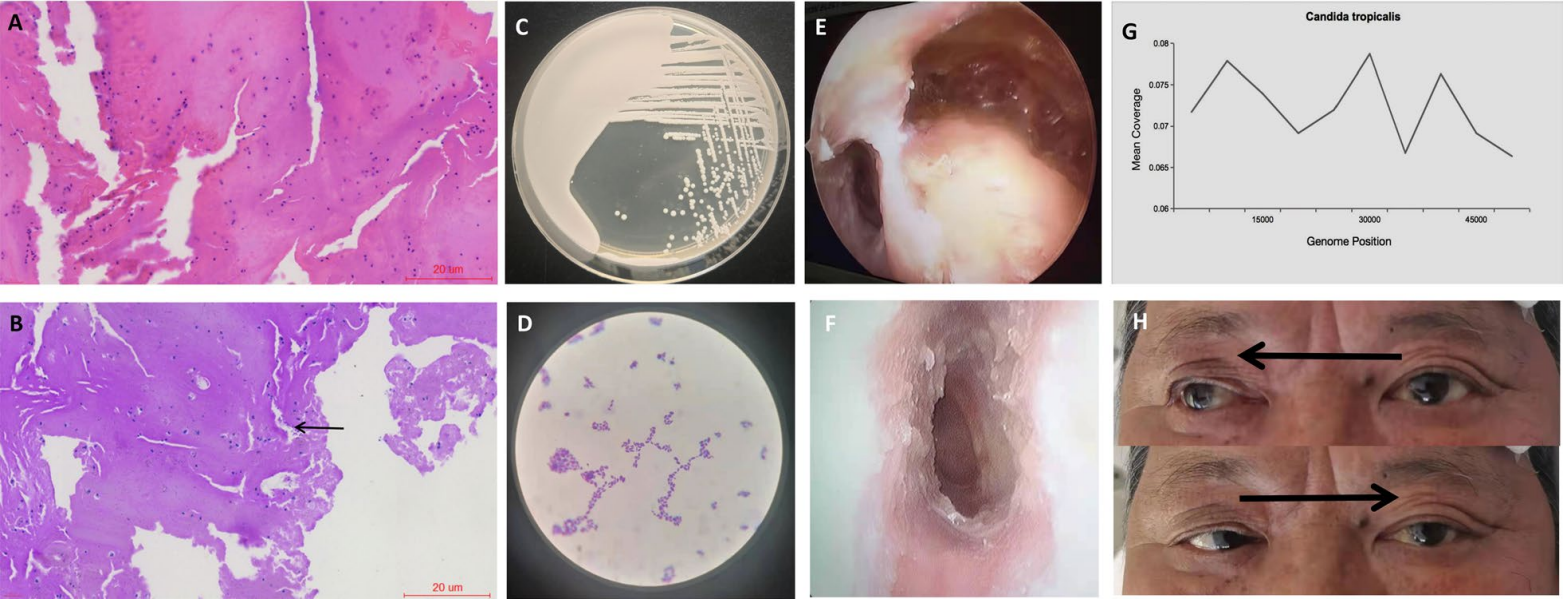
The patient's pathological section, bacterial culture, postoperative endoscopy and eye motility Fig. **A** HE staining of the mastoid granulation of the left middle ear suggested chronic inflammation of local mucosa with neutrophil infiltration. Scale bar = 20 μm. **B** PAS staining of the pathological section of the mastoid granulation of the left middle ear indicated a small amount of yeast-like fungi in the exudate of the middle ear (blue arrow), accompanied by budding phenomenon, and the morphology suggested yeast infection. Scale bar = 20 μm. Image **A** and **B** were obtained using the EasyScanner (Motic, Xiamen, China) and its associated software (Motic DSAssistant Plus, Motic). **C** Bacterial culture of secretion from left ear suggested near smooth yeast, CHROMagar candida chromogenic medium,72 h, creamy colorless smooth colony. **D** Bacterial culture of the secretion from the left ear was seen under the microscope (× 1000 times). Gram staining was positive and swarms of ovoid spores were seen. **E** Overall view of mastoid cavity and external auditory meatus under endoscopy after operation. **F** White punctate secretions of external auditory canal were observed under endoscopy. **G** mNGS showed candida tropicalis. **H** The patient had limited abduction and adduction of the left eye

Patient, male, 78 years old, was admitted to the hospital on September 18, 2021 due to "persistent discharge of pus from the left ear for 3 months, accompanied by ipsilateral earache and headache". Before admission, he had been treated continuously with ceftriaxone and metronidazole for one month in another hospital without significant improvement. There was no fever, no dizziness, no nausea, no vomiting during his illness. He has a history of chronic otitis media for more than 30 years, with recurring discharge of pus from the left ear. His left ear hearing had significantly decreased. Physical examination on his admission showed: Swelling of the left external auditory canal, existing medium-sized perforation of tympanic membrane and pulsating exudate. The discharge was a light pink viscous liquid with no odor. Audiometry test found that moderate mixed deafness in the left ear with an average hearing threshold of 65 dB. ABR showed that peripheral auditory nerve damage on the left side. Computed Tomography (CT) and enhanced Magnetic Resonance Imaging (MRI) of the middle ear showed: left middle ear mastoiditis (Fig. 1A, C, E, G). Bacterial culture of ear secretion was negative. Blood routine examination result is WBC:11.98*109/L, N:72.2%, c-reactive protein (CRP) is: 11.31 mg/L. Patient was diagnosed with " Chronic otitis media and mastoiditis with otitis externa ". Patient had been treated with antibiotic at another hospital for almost one month, but without significant improvement, so we had to drain the discharge to prevent intracranial complication. On the 4th day after his admission, radical mastoidectomy of the left middle ear was performed. During the surgery, the mastoid was found to be diploetic type with few mastoid cells and small tympanic antrum. Light pink granulation tissue was found on mastoid cells, tympanic antrum and attic. Tissue was removed and sent to pathology; Test result showed: left middle ear mastoid granulation, local mucosal chronic inflammation with neutrophil infiltration (Fig. 2A). Intraoperative cavity was filled with iodoform gauze. Patient was treated with levofloxacin and dexamethasone through intravenous injection. Headache was relieved and ear pain is gone. One week after the surgery, iodine strips were removed. Patient was discharged from hospital.

After discharge, patient’s left ear pus discharge was relieved, but left side headache increased, mainly at the top of his temporal. Patient needed painkiller to help for sleep at night. Then, 25 days after the surgery the patient was admitted again to the hospital as mouth lopsided condition suddenly showed up. We found that the left ear intraoperative cavity was dry and with some white cheese like material (Fig. 2E, F). The left mouth lopsided had air leaking, left eye was closed and weak, and the left frontal lines became shallow. Audiometry test showed: severe mixed deafness in the left ear (mean hearing threshold 90 dB). CT and enhanced MRI of the middle ear (Fig. 1B, D, F, H) showed: new soft tissue shadow in the residual mastoid cavity. So patient was diagnosed as "left middle ear mastoiditis complication with peripheral facial palsy. Blood routine examination results are: WBC 14.46*109/L, N89%, CRP 7.69 mg/L, ESR 72 mm/h. Patient was treated with cefepime anti-inflammatory, betamethasone and nerve nutrition. The headache was relieved during the steroid treatment, but his condition became worse and worse when the steroid dosage was reduced a week later, and a small amount of light yellow odorless fluid began to discharge from his left ear again.

After steroid withdrawal, dizziness, nausea and vomiting occurred on the 14th day of his second admission. His symptoms were reduced after symptomatic treatment, but diplopia reoccurred. Ophthalmic examination showed that only limited abduction of the left eye, pupil was large and round with reflection to light (Fig. 2H). MRI and CT of the head excluded cerebral infarction, cerebral hemorrhage, and tumorigenic lesions. Two lumbar punctures were performed successively and showed: intracranial infection was excluded as his intracranial pressure was normal. Cranial magnetic resonance venography (MRV) examination suggested that thrombosis was in the superior sagittal sinus (Fig. 1I), so betahistine and ginkgo biloba were infused to patient to improve microcirculation. 2nd review of the patient's middle ear CT and MRI showed that the apex of petrous part was blurred and had inflammatory changes (Fig. 1A–H), then with patient’s other symptoms of continuous headache, facial parlay, vertigo diplopia and cerebral venous sinus thrombosis. We considered that patient has otitis media complication "petrositis". Blood tuberculosis related indicators showed: Mycobacterium tuberculosis IgG, IgM and T cells of tuberculosis infection was negative. The ear secretions were taken for culture several times, and 2 results suggested: Candida glabrata (Fig. 2C, D).

**Fig. 3:**
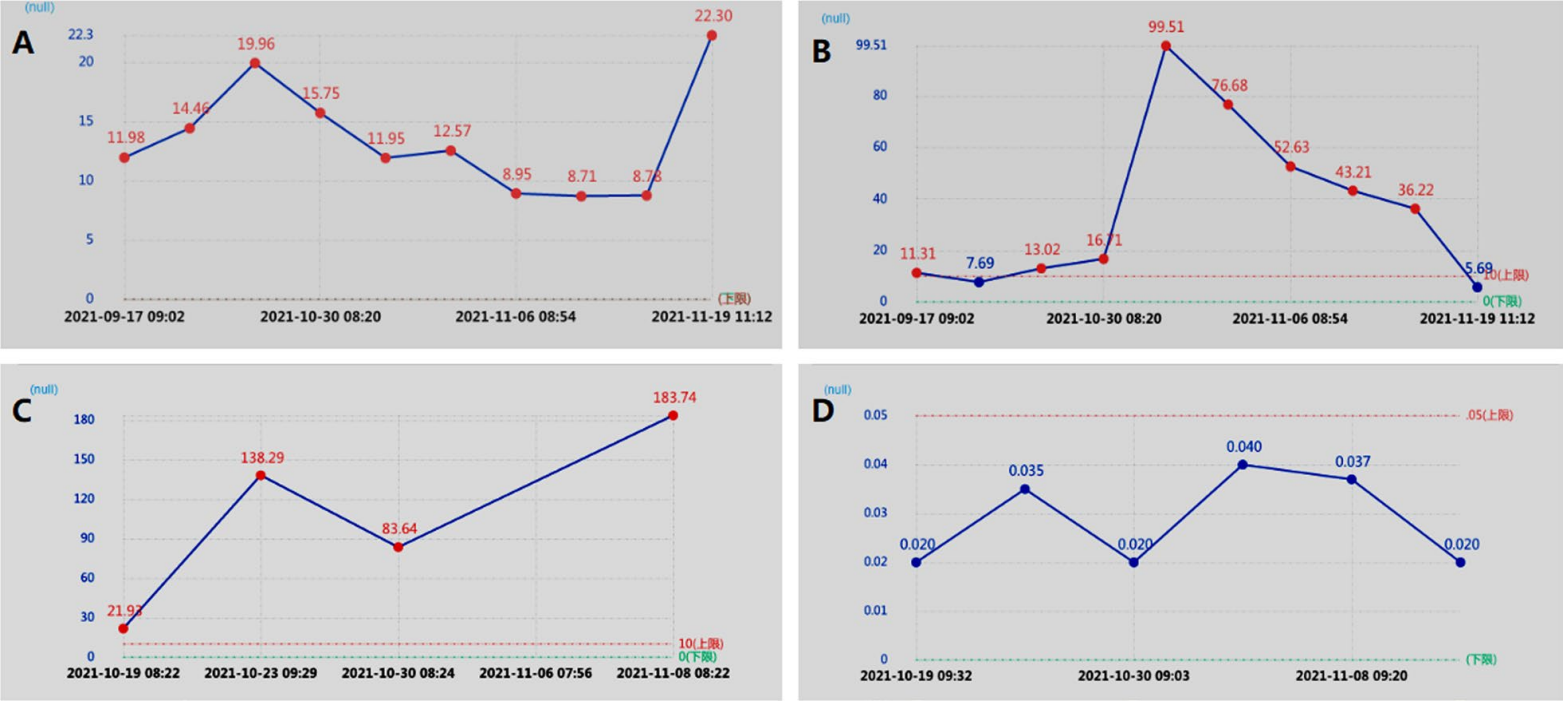
Trend chart of inflammatory indicators in patients from September to November in 2021. **A** The trend chart of leukocyte was higher than normal at the beginning of admission, decreased after vancomycin and fluconazole treatment, and increased in the later period, which might be related to hormone use. **B** C-reactive protein trends. At the beginning of admission, it was slightly higher than normal, then increased, and decreased to normal after vancomycin and fluconazole treatment. **C** Amyloid A trend map. Since admission slightly higher than normal, after the rise is obvious. **D** Procalcitonin trend chart. It's been within the normal range

**Fig. 4:**
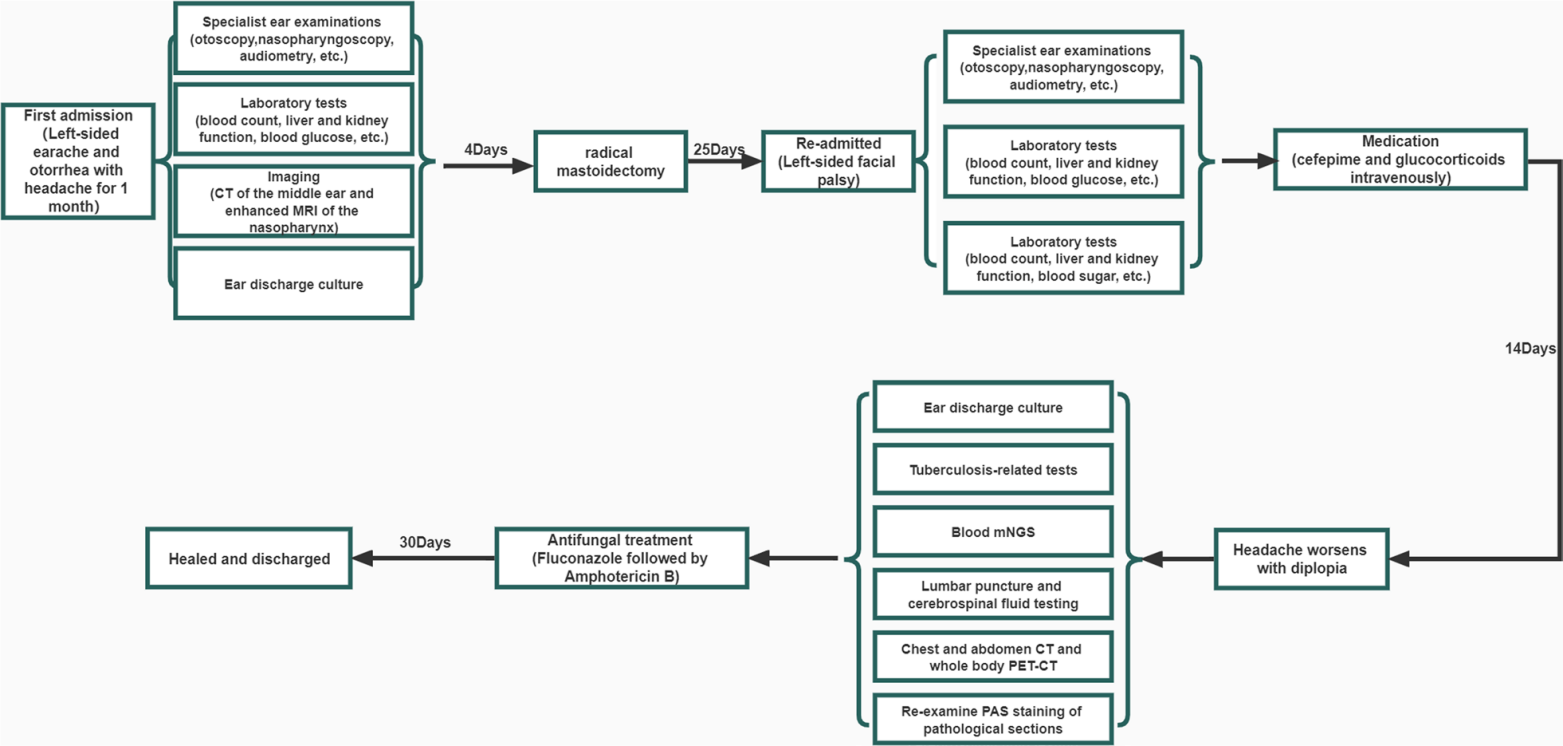
The flow chart of patient's diagnosis and treatment

The patient was tested for blood pathogen mNGS detection.Microbial cell-free DNA was extracted from blood using the VAHTS Serum/Plasma DNA Kit (Nanjing Vazyme Biotech Co., Ltd, Nanjing, China) according to the manufacturer’s instructions, High-throughput sequencing was carried out by genetic sequencer MGISEQ-200 (manufactured in 2021 by Wuhan MGI Tech Co., Ltd, Wuhan, China).The software package mNGS SmartDxTM-3.2 (developed by MedcareDx Biotech Co., Ltd, Shanghai, China) was employed to conduct bioinformatics analysis. The final result was suggested as C. tropicalis (Fig. 2G). Multiple examinations of the patient's inflammatory indicators concluded: the presence of bacterial infection. The routine blood tests showed leukocytosis with an elevated neutrophil percentage (Fig. 3A), elevated C-reactive protein (Fig. 3B) and continuously elevated amyloid A (Fig. 3C), while calcitoninogen level was always normal (Fig. 3D, excluding sepsis). Based on the above examination results, patient was infected with mixed bacteria and fungi at the apex of petrous part. So patient was infused with vancomycin combined with fluconazole for one week, but with no improvement. The headache was still severe, eyeball was still in the center (Fig. 2H) and the tongue leaned to the left. Further chest and abdomen CT and PET-CT excluded neoplastic disease. So we re-examined pathological section. PAS staining showed a small number of yeast-like fungi in the exudate of the middle ear, accompanied by budding phenomenon. This indicated yeast infection (Fig. 2B). Treatment was adjusted by stopping vancomycin but adding antifungal medication with intravenous of amphotericin B and oral flucytosine tablets. The headache disappeared after one week of new medication, the facial palsy disappeared after 2 weeks of medication and diplopia condition was reduced, then finally disappeared after 1 month of new medication. There were no unconsciousness conditions such as neck stiffness, limb mobility disorder, and projectile vomiting during patient illness and treatment. There was no recurrence after three-month follow up. We mapped the consultation process of this patient into a flow chart (Fig. 4) using the CmapTools (https://cmap.ihmc.us) software tool (Software tool) recommended by Behzadi et al. [5].

## Discussion and conclusion

### The diagnosis of petrositis and the difficulties during the diagnostic of this patient

Petrous apex syndrome, also known as Gradenigo’s syndrome [1], is a group of symptoms caused by lesions of petrous apex located at the temporal region that damage the abducens nerve and the ocular branch of the trigeminal nerve. It is a very rare clinic disease and mostly is caused by otitis media, mastoiditis and petrous apex tumor [6]. There is only one layer of dura mater between the semilunar ganglion, abducens nerve of the trigeminal nerve and the petrological apex of the temporal bone. So when the middle ear mastoiditis, cholesteatoma and petrological tumor occur at the petrological apex, these two nerves can be attacked. Main clinical manifestations are: pain in the trigeminal nerve area, loss of corneal reflex, diplopia and strabismus caused by abductive nerve paralysis, sometimes symptoms of meningeal irritation and peripheral facial paralysis [6–8].This disease usually is not primary disease in clinical practice often secondary [6–8]. Not all the classic triad symptoms are present. However, sometimes due to the severity of the disease, it may be worse than the "triad" [4]. There are other symptoms such as: ipsilateral paralysis due to involvement of the 7th cranial nerve, vertigo due to damage of the 8th, 9th and 10th cranial nerve, tongue leaning, and sensorineural hearing loss due to involvement of the inner ear labyrinth [3]. In this case, the patient first developed ear discharge, earache, and headache. With further development of the disease, facial paralysis, vertigo and diplopia symptoms showed up. However, we still need to exclude neoplastic lesions in nasopharynx, intracranial, lymphoma, tuberculosis and cerebrovascular diseases. One of the difficulties during the diagnosis of this patient was that the “triad” symptom showed gradually, with vertigo, paralysis of glossopharyngeal nerve, trochlear nerve, oculomotor nerve of many pairs of cranial nerve.

The second difficulty in the diagnosis of this patient was atypical imaging changes. CT and MRI examinations play an important auxiliary role in the diagnosis of porosities [9, 10]. MRI showed long T1 and long T2 signals in the petrous apex, which were enhanced after reinforcement. T2WI showed equal, slightly higher, or lower signals, bone damage in the lesion area; and involvement of adjacent structures including Meckel cavity and cavernous sinus. And enhanced scans showed obvious enhancement (Fig. 1E, F). Petrositis is very rare case for clinical practice. So CT imagine shows strong characteristic, it can sometime be mis diagnosed as middle ear mastoiditis. In addition, the petrous apex is a complex area with multiple variations that can be bilaterally symmetric or asymmetric [11]. This makes CT judgment more challenging. The patient’s CT image in this case showed blurred mastoid process, superior tympanum, tympanum sinus and petrous apex, with no obvious bone damage at the petrous apex (Fig. 1A, B). Although headache was present at the first admission, but patient was not diagnosed with petrositis, only at patient 2nd admission, with his symptoms of facial paralysis and diplopia, then after retrospective radiography (Fig. 1) he was diagnosed with petrositis.

The third difficulty was the patient's pathogenic bacteria. Pseudomonas aeruginosa is the most common pathogenic bacteria of petrositis, then viridans streptococci [12, 13], Fusobacterium [14] and anaerobic bacteria [15] are secondary. There are still some reports on tuberculous petrositis [16–18]. We performed relevant exam to exclude tuberculosis infection. After blood mNGS examination combined with late bacterial culture and Periodic Acid-Schiff (PAS)staining of pathological sections, candida was finally identified as the pathogenic bacteria. This is very rare. There is one fatal case of fungal otitis media complication by petrositis was reported by an author in 2021, [19]. With today's widely usage of antibiotics, fungal infection as a pathogenic bacteria need to be considered. To identify the pathogenic bacteria, we also used blood mNGS technology. This technology involves all the nucleic acid sequence in the specimen, which can cover all testing bacteria, mycoplasma, chlamydia, rickettsia, helix, fungi, viruses, parasites etc. [20]. One of the advantages of this technology is to detect culture-negative bacteria/fungal pathogens [21]. In this case, the secretion culture of the patient was negative for several times at the beginning, but by using this technology, it provided good reference (Fig. 2G). Through this case it demonstrates that Hematoxylin–eosin (HE) staining of pathological sections was not sensitive to the diagnosis of fungal infection (Fig. 2A), but PAS staining was sensitive to it (Fig. 2B). In this case, the routine blood leukocytes, c-reactive protein and amyloid A were higher than normal during entire illness (Fig. 3A–C). This misleads the diagnosis of fungal infection. The patients may have both fungal and bacterial infection coexistence. But at the end, patient was treated with antifungal drugs, sometime fungal infections can also lead to an increase in inflammatory indicators such as white blood cells level.

Another difficulty in the diagnosis of this patient was that his cranial magnetic resonance (MRV). It showed superior sagittal sinus thrombosis (Fig. 1I). The direct signs of head MRV in diagnosing superior sagittal sinus thrombosis are: losing of high blood flow signals in normal cerebral veins (sinuses) or sign of blurred and irregular low blood flow. This sign is not affected by temporal changes in the thrombus signal. This type of thrombosis can also lead to persistent and severe headache, but the patient had no typical thrombosis symptoms such as no unconscious disorder, no limb movement disorder, no intracranial hypertension (severe nausea and vomiting), no papilledema. His headache has not been reduced by simple antithrombotic therapy. Otitis media can lead to the thrombosis of sigmoid sinus and superior sagittal sinus. It can be caused by infection directly destroying bone and entering venous sinus, then resulting mural thrombosis, or by indirectly infection to the vein of the mastoid process, resulting local micro abscesses of blood vessels and infectious thrombosis [22]. In 2015, there were also reports of petrotitis complicated with cavernous sinus thrombosis and meningitis [23]. Theoretically, the patient's left otitis media should have left sigmoid sinus thrombosis first. But he had superior sagittal sinus thrombosis instead of thrombosis (Fig. 1J) so that why we considered petrotits.

### Treatment of petrotitis: surgery is risky and drug therapy is feasible

The difficulty in the treatment of this case was the options between surgery and conservative treatment, The drug therapy has been adjusted for several times. The ideal treatment of petrotitis is controversial and often depends on the severity of the clinical presence [24]. Before the invention of antibiotics, surgery was the main treatment for petrotitis. As there are two major controversies about the pathologic process of petrotitis, so different surgical approaches are suggested. Some experts believe that the petrous apex is independent of the mastoid area without small chambers of gasification, so the petrotitis is caused by local osteomyelitis [25]. The venous plexus of the internal carotid artery at petrous apex (hematogenous spread), is an important route of dissemination of inflammation. Therefore, the posterior labyrinth approach and temporal bone air-room resection and middle cranial fossa approach for abscess drainage are recommended [25]. Other experts believe that petrotitis like other gasified mastoid areas, is a kind of air-room fusion inflammation. and the air chambers around the internal carotid artery are an important route of inflammation dissemination, therefore, petrous apex resection is recommended [25]. In some recent reports, some surgeons combined transmastoid and middle cranial fossa [26]. Regardless which surgical approach, it is traumatic and high risk. Whether further reoperation should be performed for this patient who had undergone radical mastoidectomy? After analyzing the situation, we decided to use conservative drug treatment. When the patient was admitted to hospital for the first time, we had used the ceftriaxone and metronidazole for a month. And his ear secretions bacteria culture was negative. His headache was not released after using ceftazidime and levofloxacin according to experience. Patient condition was aggravated accompanied by peripheral facial paralysis and diplopia. We Adjusted antibiotics and upgraded to vancomycin combined with fluconazole, but we didn’t see any improvement. Finally, antifungal drug therapy was changed to cure to patient.

Recently, more authors have recommended the conservative treatment for non-surgical intervention and intravenous antibiotic therapy [8, 27, 28]. Plodpai et al. [29] reported a case of Gradenigo’s syndrome as secondary to chronic otitis media with previous radical mastoidectomy. This case was successfully treated by intravenous antibiotics and topical antibiotic ear drops. Some authors [30] summarized the diagnosis and treatment of petrotitis in the past 40 years and believed that antibiotics are still the main method for treatment. And Surgery is only performed for non-responding antibiotic cases. Most authors recommended to use cephalosporin antibiotics and metronidazole with or without vancomycin [30, 31]. Empirical intravenous antibiotics includes common drugs for bacterial mastoiditis (staphylococcus aureus, streptococcus pneumoniae, streptococcus pyogenes, and pseudomonas aeruginosa or anaerobic bacteria [32, 33]. In our case, ceftriaxone and metronidazole were used before admission to our hospital, then levofloxacin, ceftazidime, piperacillin, sulbactam and vancomycin were used after admission. Theoretically, if it was bacterial petrotitis, all these medications should be effective. But our case was caused by fluconzol-resistant fungus. There is no doubt that petrotitis caused by infection is equivalent to osteomyelitis, which requires intensive and prolonged antibiotic treatment to avoid recurrence [8].

### Causes of fungal infection, characteristics of yeast in this case and treatment of fungal infection (emergence of drug-resistant bacteria)

In this case, the bacteria caused the pathogenic infection is Candida, we recalled that the patient had long term chronic otitis media and repeatedly used ofloxacin ear drops then he started to have ear pain and ipsilateral headache. Two combination broad-spectrum antibiotics had been used for more than 1 month before admission, but the condition didn’t get improved and became even worse. Thus, a fungal infection should be considered. However, the initial culture of ear secretions was negative, so our diagnosis and medication were misled by the result.

*Candida* is a kind of deep infection fungi, there are many species, the pathogenic candida includes: *C. albicans, C. tropicalis and C. parapsilosis* etc. [34]. Bacteria can secrete adenosine to block neutrophils then produce and release oxygen free radicals; Aspartic protease can be produced to degrade extracellular matrix and cause tissue damage. The common drugs for treatment are fluconazole, ketoconazole, amphotericin B and itraconazole [35, 36]. And fluconazole is the first choice for the clinical treatment of candida infection. It achieves bactericidal effect by affecting the biosynthesis of ergosterol in the fungal cell membrane and changing the permeability of the cell membrane. So it is considered as first option for treatment of deep fungal infection [37]. In this case, however, the patient was infected with a drug-resistant strain that was ineffective for fluconazole intravenous infusion.

With the extensive usage of antifungal drugs in the clinical practice, the composition ratio of variants candida bacteria changes, and candida bacteria’s drug resistance increases. According to some reports: for different type of candida bacteria, their sensitivity varies greatly with anti-fungal drugs [38]. Current data shows that both Candida tropicalis and Candida krusei have high resistance to fluconazole, and this can be natural resistance. Therefore, fluconazole should be used with super caution. We must analyze the candida identification and drug sensitivity test results to guide clinical drug use [39]. In recent years, there are more cases related to infection caused by Candida tropicalis and Candida tropicalis’s resistant to fluconazole [40–42]. The main mechanism of drug resistance is the mutation of drug target enzyme gene ERG11 [43]. As target enzyme encode is changed by ERG11, so efflux pump genes on cell membrane overreacts and biofilm is formed, etc. [44–47]. The ultimate mechanism is that fungal cells mutate and create bypass pathways. To prevent cell membrane changes and accumulation of toxic products, an alternative pathway is created to avoid interruption by azole drugs, and this allows fungi to maintain cell membrane function [48]. Amphotericin B is sensitive to several common candida, but it has a large side effect on human, so it is limited on clinic practice. Generally, reduced dosage of amphotericin B is combined with azole drugs to reduce side effects [45]. In this case, amphotericin B combined with fluorocytosine was used to treat the patient and finally achieve the healing effect.

Research on the treatment of drug-resistant fungi is currently a hot topic, and one study found that P-glycoprotein (P-glycoprotein)’s efflux inhibitors P22CP and P34CP can reduce the FLC (fluconazole) values of multidrug-resistant Candida strains. This suggests that efflux activity contributes to the overall resistance of microbial strains [49]. Efflux- inhibitors, EIs) inhibiting the efflux pump can enhance the clinical effectiveness of antibiotics as their substrates [49]. So the search for efflux pump inhibitors (EIs) is the direction of research for the treatment of MDR-causing bacteria [50]. There are also studies on plant-derived products and essential oils to treat multidrug-resistant Candida, such as Ruta graveolens essential oil (REO) [51], oil macerate of Helichrysum microphyllum Cambess [52], thyme oil ( Thymus vulgaris essential oil) [53], etc., Good results have been achieved by either using alone or combining with antifungal drugs. Chitosan (chitosan) has also been found to have a significant synergistic antifungal effect in combination with fluconazole (fluconazole) [54]. It is effective against both Candida species and their resistant strains.

The first difficulty in the diagnosis and treatment of this patient was that the typical symptoms only appeared gradually slowly, and even beyond the "triad of symptoms", it also showed multiple pairs of cranial nerve involvement; The second difficulty was the initial imaging presentation was atypical and cerebrovascular magnetic resonance showed symptoms thrombosis and stenosis, led to different conclusion. The third difficulty was that the treatment with a variety of advanced antibiotics and conventional antifungal drugs were ineffective. In conclusion, when otitis media is combined with persistent and severe headache, it is important to consider the possibility of Petrositis, even the imagine analysis doesn’t obviously indicates. Conservative drug treatment is a feasible choice. Broad-spectrum with high-efficient antibiotics should be added for treatment while bacterial culture of secretions is performed. Fungal infection or even drug-resistant fungal infection should be considered, when treatment was not effective, and medication should be adjusted in time. Blood NGS tests can also provide good reference.

## Data Availability

Data sharing is not applicable to this article as no datasets were generated or analyzed during the current study.
